# Compounded trauma: A qualitative study of the challenges for refugees living with advanced cancer

**DOI:** 10.1177/02692163211000236

**Published:** 2021-03-26

**Authors:** Ping Guo, Sawsan Alajarmeh, Ghadeer Alarja, Waleed Alrjoub, Ayman Al-Essa, Lana Abusalem, Asem Mansour, Richard Sullivan, Omar Shamieh, Richard Harding

**Affiliations:** 1 School of Nursing, Institute of Clinical Sciences, College of Medical and Dental Sciences, University of Birmingham, Birmingham, UK; 2 Cicely Saunders Institute of Palliative Care, Policy & Rehabilitation, Florence Nightingale Faculty of Nursing, Midwifery & Palliative Care, King’s College London, London, UK; 3 Center for Palliative & Cancer Care in Conflict (CPCCC), King Hussein Cancer Center (KHCC), Amman, Jordan; 4 Department of Palliative Medicine, King Hussein Cancer Center (KHCC), Amman, Jordan; 5 King Hussein Cancer Center (KHCC), Amman, Jordan; 6 Chief Executive Office, King Hussein Cancer Center (KHCC), Amman, Jordan; 7 Institute of Cancer Policy, King’s College London, London, UK; 8 College of Medicine, the University of Jordan, Amman, Jordan

**Keywords:** Advanced cancer, experiences, needs, palliative care, qualitative study, refugees

## Abstract

**Background:**

Although palliative care is now an essential health service under Universal Health Coverage, ensuring access and appropriate care for refugees is a specific challenge for this large population.

**Aim:**

To identify the needs and experiences of adult refugees in Jordan with advanced cancer and informal caregivers.

**Design:**

A qualitative study using semi-structured interviews.

**Setting/participants:**

Participants were purposively sampled at two Jordanian hospitals to achieve heterogeneity by age, gender, country of origin, and primary diagnosis.

**Results:**

Twenty-nine refugees (22 patients, 7 caregivers) participated, and four themes were generated: (1) Psychological distress and sustaining social support. Refugees often experienced unmet psychosocial needs. However, psychosocial support was reported either absent or limited. (2) Knowledge and uncertainty. Lack of information and poor communication between healthcare providers and patients caused significant distress due to uncertainty. (3) Family anxiety and support roles. Being away from the home country cut patients and caregivers off from their wider social support network, which added increased anxiety and responsibilities to caregivers. (4) Compounded trauma and poverty. Many refugees have experienced trauma related to war that may affect their physical and mental health. They faced serious financial crises caused by the rising cost of medicines and treatment.

**Conclusions:**

This study reveals the impact of fractured families and networks on social support in advanced cancer, and the compounding trauma of the disease for refugees. Detailed person-centred assessment and emphasis on psychosocial support is essential, and home-based care should not presume community support for patients to remain at home.

**What is already known about the topic?**

- Refugees, as a minority group in any given society, experience many stressful and traumatic situations prior to, during, and after transfer, and a high burden of physical, psychological and socioeconomic challenges.
- Understanding the experiences of displacement, humanitarian and protection needs and access to appropriate solutions (e.g. healthcare, education and shelter) among refugees is important but has not received sufficient attention.
- Evidence reveals a scarcity of data on palliative care needs and interventions provided in crises, challenges of care provision particularly due to inadequate pain relief resources and guidelines, a lack of consensus on the ethics of providing or limiting palliative care as part of humanitarian healthcare response, and the importance of contextually appropriate care.

**What this paper adds?**

- Refugees with advanced cancer and caregivers often experienced unmet psychosocial needs, and lack of information and poor communication between healthcare providers and patients caused significant distress due to uncertainty.
- Being away from the home country cut patients and caregivers off from their wider social support network, which added increased anxiety and responsibilities to caregivers.
- Many refugees have experienced trauma related to war that may affect their physical and mental health, and they faced serious financial crises caused by the rising cost of medicines and treatment.

**Implications for practice, theory or policy**

- This study reveals the impact of fractured families and networks on social support in advanced cancer, and the compounding trauma of the disease for refugees.
- The data reveal challenges in obtaining social support in the community and emphasise the need to better understand homecare needs and models for this population.
- Effective communication and interactions between staff and patients and their families and appropriate psychosocial services must be considered essential components of palliative care to address the unmet needs of this vulnerable group.

## Introduction

Jordan hosts the second-greatest refugee to citizen ratio of any country in the world.^1,2^ In 2018 there were 2,175,491 refugees from Palestine, 661,859 from Syria and 66,262 from Iraq displaced to Jordan.^3^ The United Nations Relief and Works Agency (UNRWA) reports a significant number of Palestinians in Jordan who are not registered and are therefore considered to be ‘displaced persons’ rather than refugees. Syrian refugees alone account for 10% of Jordan’s population, and the majority of these live outside refugee camps in Jordanian communities,^4^ placing considerable strain on access to basic services.^5^

Refugees, as a minority group in any given society, experience many stressful and traumatic situations prior to, during, and after transfer,^6^ and a high burden of physical, psychological and socioeconomic challenges. Iraqi refugees in Jordan report unemployment (90.0%), poverty (91.4%), anxiety and depression (42.0%), fear and insecurity (8.7%), isolation (6.6%) and high blood pressure and diabetes (32.9%).^7^ Syrian refugee families in Jordan report a number of challenges and consequences that are legal (service access rights) and civil (documentation of birth, marriage and death registration).^8^

Understanding the experiences of displacement, humanitarian and protection needs, and access to appropriate solutions (e.g. healthcare, education and shelter) among refugees is important but has not received sufficient attention.^9^ In Jordan, although the United Nations High Commissioner for Refugees (UNHCR) and its health partners offer some health services to registered asylum seekers and refugees, only Syrians and Iraqis benefit from subsidies at government facilities. All others are required to pay elevated foreigner rates at Ministry of Health facilities, which prevents many from accessing essential services for urgent and severe problems.^9,10^ Projections using the World Health Organisation (WHO) mortality data in upper middle-income countries (such as Jordan) suggest an 88% increase between 2016 and 2060 in people facing serious health-related suffering at the end of life.^11^ The greatest increase will be in cancer for these countries (96%), and cancer is currently the second leading cause of death in Jordan (15%).^12^ Cancer care for refugees in Jordan bears an estimated annual cost of 2.09 million euros.^13^ Refugees and immigrants have poorer access to cancer care than other residents of the Middle East.^14^

Palliative care is an essential component of any humanitarian response, however, there is limited evidence about the needs and experiences of palliative care and symptom relief among refugees with advanced illness and their caregivers. A cross-sectional study^15^ of refugees with serious health problems (such as significant physical disabilities, treatment-resistant tuberculosis, cancer, HIV infection) and caregivers living in Rohingya refugee camps in Bangladesh highlighted that many refugees with serious health problems experienced significant physical, emotional and social suffering due to a lack of access to pain and symptom management and other essential component of palliative care. Caregivers reported an average of 13.8 h of care per day and suffered sleep difficulties, lack of appetite and lack of pleasure in life in relation to their caregiving role. It is recommended that humanitarian responses should develop and incorporate palliative care and symptom relief strategies that address the needs of all people with serious illness-related suffering and their caregivers including refugees.

Palliative care supporting people with serious illness or those nearing death have been excluded in humanitarian emergency and crisis response until recently.^16^ A systematic review of the literature on palliative care in humanitarian crises revealed a scarcity of data on palliative care needs and interventions provided in crises, challenges of care provision particularly due to inadequate pain relief resources and guidelines, a lack of consensus on the ethics of providing or limiting palliative care as part of humanitarian healthcare response, and the importance of contextually appropriate care.^17^ More research and open discussion on palliative care in humanitarian crises are needed.

Palliative care is not routinely available across Jordan.^12,18^ Given the unique challenges of refugees, particularly those living with advanced cancer, and the scant research attention to date in this field, this study aimed to identify the specific experiences and palliative care needs of refugees in Jordan with advanced cancer and their informal caregivers.

## Methods

### Design

This was a qualitative cross-sectional study which used semi-structured interviews and thematic analysis, and is reported in accordance with the consolidated criteria for reporting qualitative research (COREQ) guidelines.^19^ This study was approved by the Research Ethics Committee at King’s College London (HR-17/18-7243), King Hussein Cancer Center (18KHCC70) and Jordanian Ministry of Health (MOH REC 1800110).

### Setting and participants

The study was conducted in two sites in Amman, Jordan: King Hussein Cancer Center (KHCC) and Al Bashir Hospital. KHCC provides comprehensive cancer care for about 60% of cancer cases in Jordan, and patients from several Arab nationalities. Its palliative care unit provides inpatient, outpatient and home-based care. Al Bashir Hospital is a public hospital governed by the Ministry of Health and provides supportive care. The supportive care in the oncology service at Al Bashir Hospital offers inpatient and outpatient pain and symptom management to cancer patients, in the form of consultation service. However, the limited staff and resources pose a challenge to identifying and addressing comprehensive palliative needs of patients.^20^

Adult refugees (⩾18 years) with advanced cancer receiving care from the study sites and informal caregivers were eligible to participate in this study. Informal caregivers are defined as ‘unpaid, informal providers of one or more physical, social, practical and emotional tasks. In terms of their relationship to the patient, they may be a friend, partner, ex-partner, sibling, parent, child or other blood or non-blood relative.’^21^ We excluded those with severe physical or psychological problems considered by clinicians too ill to be approached, and those unable to communicate in Arabic or English. Participants were sampled purposively to achieve heterogeneity in age, gender, country of origin and patient’s primary diagnosis. Caregivers were recruited independently of the patients and interviewed separately.

All clinicians who were treating patients during the study period at the two sites (i.e. oncologists, palliative care doctors and nurses) received the specific training including the eligibility criteria, and how to assess and identify potential participants. Eligible patients including both inpatients and outpatients were identified and provided with information sheets by treating clinician. Those interested in participation were approached by dedicated research staff who provided further details and answered any questions. The outpatients were approached while waiting for, or after, their consultation with treating clinician. Home care patients were not approached and included in this study. Caregivers were approached and consent sought similarly. All potential participants were given 24 h or longer to consider if wished. Those who decided to participate gave written consent (or an ink thumb print if unable to write through disability, weakness or illiteracy). The information sheets and consent forms were translated into Arabic using forward/backward translation to ensure consistency.

### Data collection

Face-to-face, semi-structured in-depth interviews were conducted by research staff from KHCC in the participants’ preferred settings between 1st October 2018 and 31st May 2019. The interviewers (SA, WA and LA) had extensive experience in palliative care and qualitative research. However, no relationship existed at the time of consenting and interviewing participants. A topic guide was developed from a review of evidence on experiences of displaced persons in Jordan,^7–10^ palliative care needs in humanitarian emergency and crisis settings,^16,17^ and refined by the wider cross-national research team.

The patient participants’ topic guides addressed (1) the experiences of living with terminal illness, facing end of life and family support; (2) personal priorities, needs and concerns, (3) distress and challenges as refugees and coping strategies; (4) palliative care needs and experiences of refugees with advanced cancer (e.g. family needs, religious/spiritual needs, trauma and displaced experience, preferences for advance planning, decision making and communication); (5) what would make care more patient-centred, and how should that be delivered. Caregivers were also asked about their own needs/concerns and how they were met, experiences of caring for someone with advanced cancer and views towards quality of care. Data collection continued until data saturation was reached (i.e. no new themes were identified in line with the study aim). All interviews were conducted in Arabic and digitally audio recorded, anonymised, transcribed verbatim by a Research Assistant (GA), and translated into English by another Research Assistant (AA) in Amman, Jordan. The transcriber and translator were not involved in data collection. Field notes were made during each interview.

### Data analysis

Data were jointly coded by the researcher in the UK (PG) and Jordan (SA), and analysed using inductive thematic analysis in QSR NVivo 10. The two authors independently examined seven transcripts to develop a coding framework and applied the coding framework to the analysis of the remaining transcripts. Transcribing, translating, and analysing the data occurred simultaneously as the data collection proceeded to refine the topic guide. Interview data were categorised and compared enabling identification of common themes and sub-themes in the dataset. A list of themes and sub-themes were created, then examined for overlapping themes merged under descriptive labels and themes containing few quotations. Throughout this process, the data were consistently analysed to gain insight into relationship between themes.

Emerging themes were discussed between researchers to improve confirmability and dependability of the findings. The findings were interpreted within the context of the existing literature. The COREQ guidelines were used throughout the study design, data collection and analysis process to enhance trustworthiness and methodological rigor.

### Project management

The research team maintained integrity, patient privacy and confidentiality of data throughout the study. The Project Steering Committee members from both the UK and Jordan met on a regular basis to monitor recruitment, review the detailed progress of the study and make recommendations for the overall direction and strategy of the study.

## Results

### Sample characteristics

Interviews were conducted with 29 refugees (22 patients with advanced cancer, 7 caregivers) (Table 1). Interviews varied from 12 to 97 min (Mean = 58 min). Patients had an average age of 55 years (range = 26–75), with five different countries of origin (Syria, Iraq, Libya, Yemen, Palestine). Caregivers were not related to any of the patients in this study.

**Table 1.** Sample characteristics (*n* = 29).

	Patients (*n* = 22)	Caregivers (*n* = 7)
Mean age in years (range)	55 (26–75)	42 (31–55)
Gender		
Male	9	5
Female	13	2
Cancer stage		
III	5	1
IV	17	6
Cancer primary		
Breast cancer	9	1
Colorectal cancer	3	1
Gastric cancer	2	0
Pancreatic cancer	1	0
Parotid gland adenoid cystic carcinoma	1	0
Prostate cancer	1	0
Lung cancer	2	1
Myelofibrosis	1	0
Ovarian cancer	1	0
Rectal cancer	1	0
Sarcoma cancer	0	1
Gallbladder cancer	0	1
Bladder cancer	0	1
Brain tumour	0	1
Country of origin		
Syria	11	3
Iraq	4	0
Libya	4	1
Yemen	1	1
Palestine	2	2

Four themes were generated: psychological distress and sustaining social support, knowledge and uncertainty, family anxiety and support roles and compounded trauma and poverty.

### Psychological distress and sustaining social support

#### Psychosocial needs and support

Majority of patients feel frustrated, upset and desperate when they heard they had cancer, and were additionally distressed by having to stay in a Jordanian hospital. Being far away from their home country, family and social networks meant they could not provide what their children or other family members need because of the disease. However, they tried to hide their feelings to avoid hurting their loved ones.

*“I feel depressed. I scream. I feel beaten on the inside. I fake happiness in front of them because I don’t want them to be sad, but I feel sad on the inside.”* (Patient age 45, female, Syrian, Breast Cancer IV - PAL0037)

*“Sometimes I would feel sad and down, but not to the extent of depression, not the way that affects those with me at home. They all suffer with me, they want to help me, they are all there for me, so I don’t want to make it harder for them.”* (Patient age 43, female, Iraqi, Breast Cancer III - PAL0040)

A few caregivers also noticed that the patient stayed away and did not express their feelings and saw a key task to be maintaining patient morale.

*“He looks sad, but he doesn’t show it. He would sit alone sometimes. When we ask him why he is sad, he says it is nothing. But we know how he was before and how he is now after his diagnosis.”* (Caregiver age 36, male, Syrian, son caring for his father - CAL0002)

*“The doctor should try to lift his spirit. I would ask the doctor to order a blood test to check on his immunity, and even if it was poor, try to lift up his spirit. A cancer patient’s life is all about spirit.”* (Caregiver age 33, male, Palestinian, son caring for his father - CAL0014)

Family support was of crucial importance:

*“When my husband, mother-in-law or sister-in-law support me, I feel like I matter to them. It is nice to have therapists, but I will only see them for a while, but when it is family, you can feel when they’re there for you, even if it is by a phone call, you can feel the love.”* (Patient age 43, female, Iraqi, Breast Cancer III - PAL0040)

*“His family calls us from Iraq. They keep telling me not to worry and that I am fine. It makes me optimistic and I don’t let despair get into my life. They don’t leave me alone for one second. My friends come to my house to visit. . . they support me and comfort me.”* (Patient age 43, female, Iraqi, Breast Cancer III - PAL0038)

Community support was also identified as important:

*“She is our neighbour and is sick too. We comfort each other. She lifts up my spirit and I lift up hers. We understand each other. When she is sick, I tell her to forget about the disease and eat, and she says the same to me when I am sick. We help each other. Once we pour our cups of coffee, the illness is out of our thoughts.”* (Patient age 45, female, Syrian, Breast Cancer IV - PAL0037)

Even though most participants believed that clinicians should care for all aspects of the patient’s health (physical, psychological, social and religious aspects), appropriate psychological and social support was reported either absent or limited.

*“I was in so much pain and no one would listen to me, they would just give me the painkiller. There was that night where I saw death with my own eyes, I was vomiting a lot, and I told them about it, and they told me to wait for my doctor to come, that was at 2AM and I had to wait till 7AM, I died a million times. . .”* (Patient age 49, male, Syrian, Gastric Cancer III - PAL0046)

*“The patient’s mood should be cared for by more than one person, like parents, siblings and friends. The same applies for the hospital, where it’s on the whole staff, doctors, nurses, even the housekeeping staff.”* (Patient age 26, female, Libyan, Ovarian Cancer IV - PKH0021)

Family caregivers identified clinical teams as being potentially well-positioned to provide emotional support.

*“Maybe she (the patient) would listen to him (healthcare staff) more than she listens to us. We’re her children, so she might think we don’t know these stuff, but if someone specialised in therapy or spiritual counselling, she could listen to him better.”* (Caregiver age 31, female, Yemeni, daughter caring for her mother - CKH0008)

However, some participants questioned the clinical role, reporting that psychosocial-spiritual support is ‘*outside of the doctor specialty*’ (CAL0017, son caring for his father). They felt that the healthcare staff ‘*just care about the disease and don’t care about other aspects of care*’ (PAL0009; PAL0039). One patient said, ‘*I don’t blame them, they are very loaded with work, they treat me well but they can only handle that much*’ (PAL0037).

#### Spiritual care

Majority patients had strong faith in God and believed that everything is planned by God, that they should fulfil their duties towards God, pray and leave things to God.

*“As long as God is with me I have no concerns. I just want him to grant me health and energy. My body is broken. I hope God will cure me and return us to our country safe and sound.”* (Patient age 66, female, Syrian, Breast Cancer III - PAL0002)

*“I have one single certainty in my head, one that I am not willing to bargain, which is death. It is a debt we all have to pay, it has nothing to do with disease, just because I got sick doesn’t necessarily mean I will die, that’s why I take medications and try to create causes for cure, but I know this medication won’t make me live longer than I am destined to. . . my day is known, it is written, the exact date and time are written, I won’t die a minute later or a minute earlier.”* (Patient age 71, male, Palestinian, Prostate Cancer IV - PAL0045)

*“I believe in what God said in the Quran; ‘And we send down of the Qur’an that which is a cure and a mercy to the believers’. It is all about believing. If you believe the Quran will cure you, then it will. . . We do what we can do and leave the rest to God. I don’t believe it is serious because I have faith in God that there isn’t a disease without a cure. I know a stage 4 cancer is serious, but I know God’s mercy exceeds all.”* (Caregiver age 55, male, Palestinian, father caring for his son - CAL0013)

### Knowledge and uncertainty

Most patients explained that healthcare providers and family tended not to discuss diagnosis, prognosis and care plan with them, which caused significant distress due to uncertainty.

*“I wish they would tell me everything and not keep anything from me. Because when you don’t know anything about yourself, you’re always afraid and you keep thinking. . . The doctor talks to my husband and son. They are more informed about my illness. Sometimes when the doctors tell them things and they keep it a secret from me, I feel scared.”* (Patient age 45, female, Syrian, Breast Cancer IV - PAL0037)

*“At Islamic hospital, I had a feeling that the doctor wanted to keep something from me, so I told him not to, and to tell me if it was cancer. I told the doctor there was no need to ask me to wait outside and tell my son. . . Some families ask the doctor not to tell the patient about cancer, I know people who died without knowing they had cancer.”* (Patient age 55, male, Syrian, Pancreatic Cancer IV - PAL0041)

*“He (the doctor) should have explained to him that such disease seems like it is cured but it really isn’t, my father is the first cancer patient in our family so it is a new experience for us, he should have instructed him about psychotherapy and chemotherapy. The patient must be fully informed. . . Information was very scarce, they just gave him tips and headlines, if this part of your body hurts then take this pill and that’s it.”* (Caregiver age 41, male, Syrian, son caring for his father - CAL0017)

Even though most of the patients expressed their willingness of knowing their conditions, the caregivers often did not want to tell the patients the truth to protect them.

*“He asked the doctor about the report. When he told him it was good he felt relieved. Last time he spent fifteen minutes asking the doctor. I went out and came back and he was still asking him. I didn’t know what he was asking about. He wants to know what would happen if it spread or what symptoms it might cause. . .”* (Caregiver age 36, male, Syrian, son caring for his father - CAL0002)

*“He has a malignant mass in his bladder. We didn’t want to tell him about it because his spirit is really low and he gets anxious and worries about himself and his family. . . He is always anxious, so we kept it a secret to keep his spirit high.”* (Caregiver age 33, male, Palestinian, son caring for his father - CAL0014)

A minority would prefer not to be given full information to keep positive feelings, especially in relation to prognosis. However, they acknowledged the benefit of having complete information.

*“The doctor said it might recur after two years, or even after five years, so it is hard to hear that. He should have told me the treatment would stop this disease and not to think about it, but to tell me I have two years, that’s really tough. It destroyed me. The doctor should focus more on the positive and never tell the patient the negative stuff. . . It could be for the benefit of the patient to know everything, it is like knowing if the road you’re taking is jammed with traffic or not. . .”* (Patient age 71, male, Palestinian, Gastric Cancer IV - PAL0039)

*“I feel positive when they tell me the disease is regressing. They give me hope. The patient’s morale is very important, it helps the patient control the disease, but if the patient is depressed all the time, the disease will end up controlling the patient.”* (Patient age 55, male, Syrian, Pancreatic Cancer IV - PAL0041)

Patients who did not prefer to be given full information advocated for incremental or specifically timed communication of steps.

*“It’s their right to know everything about their condition, but not when they’re not in the proper psychological shape. If the patient’s mental state improves, their physical state improves too, and only then can the doctor talk to the patient honestly about their condition. It is better if the doctor doesn’t break any bad news to the patient when they’re depressed.”* (Patient age 26, female, Libyan, Ovarian Cancer IV - PKH0021)

*“A doctor can kill a patient. . . mentally, drive him through depression and mental illness. Not every patient can tolerate news like this. It depends how strong they are. The doctor can keep it a secret from the patient. I advise the doctors not to inform the patient from the beginning about their disease because the patient would be in a bad shape no matter how strong their faith is. The patient’s family can handle this and try to boost his morale, but the news has to be broken to the patient gradually.”* (Caregiver age 55, male, Palestinian, father caring for his son - CAL0013)

Other patients expressed the complications that came with incomplete information.

*“He [the doctor] should have put me in the picture, he should have told me that they might have to remove the whole kidney. He told me after they sent it to pathology that it was one of the most aggressive types of cancer. . . That was the only useful piece of information that he gave me. He did not explain the treatment plan, and tell me about surgery, radiotherapy and chemotherapy beforehand. . .”* (Patient age 49, male, Syrian, Gastric Cancer III - PAL0046)

Due to poor communication and lack of information from health care staff, patients often felt uncertain about their disease and treatment which significantly increased their anxiety level.

*“Having sufficient information would make a huge difference. It keeps me prepared for what would happen, I would tolerate those symptoms better. Without this information, it makes the patient more anxious or stressed out. . . Since it (red spots) came back again, I am worried it might come back a third time. I didn’t know what to do, should I go back to him, or see a dermatologist?* (Patient age 43, female, Iraqi, Breast Cancer III - PAL0040)

### Family anxiety and support roles

Family caregivers expressed impact on their own wellbeing,

*“It has a psychological impact, of course, when you see your own son not able to go to the bathroom, you’ll feel bad. When he is a young man at that age and to be this disabled, it kills me to see my son like that. . .”* (Caregiver age 55, male, Palestinian, father caring for his son - CAL0013)

This creates a conflict in managing the caregivers’ own emotions when trying to support family members,

*“He’s a 23-year-old boy and my daughter’s fiancé, surely that would make me upset. I felt sorry for my daughter. . . I didn’t tell her that the cancer had spread in his entire spine. . . I’m not fine myself. It hurts me to see how he was and how he is now. I cry a lot, but not in front of him. . . I try to pull myself together in front of his mother. His poor mother is crying all the time and I try to console her. There is nothing we can really do.”* (Caregiver age 54, male, Syrian, father-in-law caring for his son-in-law - CKH0007)

Poverty in the face of expensive treatment caused greater emotional pain,

*“We used to be a happy family. We would sit together and eat. We would joke around and laugh and help each other against life, but my disease affected the family. . . I have a 16-year-old who is always sad. He looks like he’s 100 years old. He prays to God to take his life, because I am sick and he can’t afford to treat me, and he is afraid he might lose me one day.”* (Patient age 45, female, Syrian, Breast Cancer IV - PAL0037)

Being away from the home country cut patients and caregivers off from their wider social support network,

*“It is very different from being in my home country. All the burdens on my shoulders here. It would have been shared with my brother, my sister, my cousin, his uncle, his mother. . . they would’ve helped me taking to and from the hospital. If I felt upset they would try to cheer me up. But here I’m all alone and all this load is on me.”* (Caregiver age 54, male, Syrian, father-in-law caring for his son-in-law - CKH0007)

### Compounded trauma and poverty

The war in the home country of the patients made their cancer treatment unavailable in their home country.

*“The situation back home is not that good. The war and everything. I did get imaging and had my eyes checked in Yemen. They told me it recurred. It is a blocked road and there aren’t any painkillers. I didn’t receive any treatment in Yemen as they don’t have the laser device. I had to come here for the laser. They told me they couldn’t treat me there, and couldn’t do the surgery or radiotherapy. If everything was available in our country I wouldn’t have come here.”* (Patient age 31, female, Yemeni - PKH0036)

*“Of course, I do want to return to my home country, but only when I am cured because the country is still unstable, and medications aren’t as available there as they are here.”* (Patient age 68, male, Syrian - PAL0007)

Traumatic experience made the whole family fall apart.

*“What could his mother do? Two of her children passed away. One of her sons fled Syria, he was married with children. He got arrested then it turned out he was dead. His wife and children are here. She also has an older son who was arrested then turned out to be dead. My brother died also before he came here. All of this happened in a short period of time. . . If there was a glimpse of hope, I would never feel tired. I am willing to go to the hospital for my entire life (to care for him), but there isn’t any hope.” (*Caregiver age 54, male, Syrian, father-in-law caring for his son-in-law - CKH0007)

*“. . .Not keep him attached to tubes and bags. My father is a practical man, if he is annoyed by kids, he goes out for a walk, and he is willing to walk for 3 hours or to buy some bread. Imagine a man like that tubed up and carrying a urine bag, it is like your sentencing him to death.”* (Caregiver age 33, Male, Palestinian, son caring for his father - CAL0014)

Many refugees applied for financial assistance from the UNHCR but faced serious financial crises when their applications were rejected. They could not afford expensive medications, imaging, treatment and even living costs. The rising cost of medicines and treatment raised concerns regarding high financial burdens.

*“I am afraid that one day I won’t be able to buy my pills. . . I wish they would lower the price of the medication or just give it to us for free. We don’t want money, just give us the drug for free. National hospitals in Syria provide chemotherapy and radiotherapy free of charge, whereas in Jordan we have to pay for it.”* (Patient age 66, female, Syrian, Breast Cancer III - PAL0002)

Some patients found much relieved financially when their applications were accepted for an exemption from the hospital. The majority of patients have been receiving treatment on their own expense for months or years. Unfortunately, they have spent everything on their treatment and could not afford any longer. It was too expensive for them to stay in Jordan for further treatment (e.g. PKH0036; PAL0038; PAL0041; PAL0042; PKH0028).

*“I have 6 daughters and 2 sons, with me and my husband. We are 10, and I have been receiving treatment for 4 years at my own expense over 12K, half of them are debts. . . This cycle is probably the last one I am gonna take because I can’t afford any more. I can barely get my children bread. . . I went to UNHCR and they told me funding was stopped especially for those who came in 2014 like us. They told me those who came in late are not funded.”* (Patient age 45, female, Syrian, Breast Cancer IV - PAL0037)

People lost their jobs due to their disease and treatment and described their financial situation in Jordan is not just rough but very rough (PAL0006). They tried to apply for Jordan Health Aid Society (JHAS) four or five times and there was no success or a long waiting time for the approval to get through. They often called their relatives for help.

*“We have a relative in Syria who covers the expenses. He sends us money, and we registered at JHAS international and they told us we have to wait for our turn until they submit our papers to the organizations. Our priorities are the colostomy bag and those cycles which cost us 80 JD each. Sometimes we can’t get those because every trip here costs us around 300 JD.”* (Caregiver age 36, male, Syrian, son caring for his father - CAL0002)

Their financial status played an important role in not only treatment, but also affected their ability to travel, pay rent, buy food, all of that. They experienced symptoms and discomfort from their disease and at the same time, catastrophic financial difficulties because of healthcare payment, which made them feel struck by lightning. As some patients described,

*“My current financial status is non-existent. . . What do we -refugees- have? Our houses are rented. We buy our water. We buy our food. What do we have? Nothing.”* (Patient age 66, female, Syrian, Breast Cancer III - PAL0002)

*“Can you imagine that my 12-year-old son and 14-year-old daughter can’t read or write? We don’t have money to find someone to teach them. . . My financial status is below zero. We rented two rooms in a house that leaks when it rains and has no heating.”* (Patient age 45, female, Syrian, Breast Cancer IV - PAL0037)

## Discussion

### Main findings

This study highlights the impact of fractured families and social networks, and compounded trauma on refugees with advanced cancer and caregivers in Jordan. Effective communication and interactions with the patients and families about their treatment and care plan and appropriate psychosocial services have been identified by the participants as essential in their care but these aspects of care are often neglected. Financial difficulties have become a major concern of both the patients and caregivers.

It is crucial to early identify and address the unique multidimensional needs of refugees with advanced disease and their family caregivers, particularly psychosocial and spiritual needs. The assessment tools have been used routinely at KHCC to expand the capacity of the clinicians to assess the needs of the patients including the Edmonton Symptom Assessment System (ESAS)^22^ for all encounters in palliative care and Distress Thermometer^23^ for all patients admitted to the Center. Currently, an initiative is underway to adapt, validate and implement the Integrated Palliative care Outcome Scale (IPOS)^24^ to reflect the specific concerns of refugees at KHCC and Al Bashir Hospital. The brief assessment tool – IPOS can help to not only identify symptoms, but also extend to capture communication and information needs, practical concerns, anxiety or low mood, family anxieties and overall feeling of being at peace. These demonstration centres will drive improvement in assessment and care of patients and families (including refugees) with palliative care needs. This work will be published separately.

Compounded trauma in refugees’ lives has been considered as a consequence of multiple historical, social and political constraints which are embedded in their personal experiences as refugees.^25^ This is echoed by the findings of our study which emphasised the importance of having a holistic understanding of the multidimensional needs unique to refugees. Our participants described a number of specific traumas, that is, forced to flee their home countries to escape serious human rights abuses and prolonged physical and emotional distress (e.g. exposure to wars and conflicts, ongoing physical, sexual and psychological oppression). Seeking a place of safety took them away from their family and social support network, which they would usually have drawn on to cope with the anxiety and stress of their cancer. When developing and evaluating complex interventions for this particular group of refugees with advanced cancer, the healthcare providers should take a person-centred approach to respecting the diverse beliefs, values and unique experiences and multidimensional needs of refugees. Person-centred care shifts away from healthcare providers deciding what is best for a patient to putting people at the centre of care and taking into account their values, preferences and needs, as an expert of their own experience. The person and their family become equal partners not only in the planning of their own care and support, but also in the design and delivery of services. This approach can improve both the experience and quality of care by ensuring people to receive holistic assessment and care, have access to appropriate care when and where they need it, and get all the information they need in a way that is accessible for them to make informed decisions for their care and support. For example, when treating and caring for Muslim refugees with advanced illness, their religious beliefs and preferences at end of life must be reflected.^26^

Policy makers and health providers in Jordan have experienced enormous challenges due to the lack of quality cancer prevalence data to inform innovative interventions and care delivery for the refugee population, and insufficient health resource allocation to support services. A qualitative interview study conducted with health officials and healthcare workers from the Jordanian Ministry of Health, multilateral donors and international non-governmental organisations identified key barriers such as limited access to international funding for the host country, the absence of long-term funding schemes, and barriers to coordination between institutions and frontline clinicians.^27^ Although health care coverage of refugees varies by host country, the UNHCR is the major payer. The UNHCR does not cover the expenses of many chronic diseases such as end-stage renal disease.^28^ In 2013, an interagency report documented a rise in the number of Syrian refugees presenting with cancer to Jordanian health clinics, of which many were children with leukaemia. Acknowledging that the cost of treatment exceeded UNHCR’s referral budget at that time, the agency looked to the specialist KHCC in Amman Jordan, and its goodwill fund, for assistance.^29^

Now is time to support the health professional training and provision of holistic palliative care interventions in humanitarian emergencies.^30,31^ Pinheiro and Jaff conducted a qualitative interview study with 21 Syrian refugees in Jordan with life-limiting conditions such as cancer, diabetes, chronic disability and renal failure and four caregivers caring for refugee children with similar conditions.^32^ This study identified the gaps and challenges of addressing the health needs of Syrian refugees with life-limiting conditions in Jordan and found that patients in refugee camps and communities would benefit from receiving palliative care services that are often either unavailable or inaccessible. Given that most Syrian refugees live outside of camps, they emphasise the need to improve national systems that will benefit all persons, by increasing national capacities to provide palliative care integrated into the existing health system in Jordan and available to everyone including nationals and refugees alike. The key findings from our study are consistent with the palliative care needs from previous studies.^15,32^ However, our study further explored the impact of fractured families and social networks, and compounded trauma on refugees with advanced cancer and caregivers in Jordan.

Much of the research on refugees has focused on their movement from non-Western countries to Western countries where immigration policies have been developed, such as the US, Canada and Australia.^7^ Middle Eastern countries such as Jordan are also coping with the arrival of a large number of refugees from neighbouring countries and managing the ensuing protection efforts. This global refugee situation poses a challenge to the knowledge base and skills required by health care providers to respond to the needs of individuals and to address the broader humanitarian and political issues.

### Strengths and limitations

To our knowledge, this is the first qualitative study to gather the first-hand information about the real experiences and needs of refugees with advanced cancer and family caregivers in Jordan. However, this study was limited by a small sample of family caregivers (*n* = 7). More caregivers may have resulted in greater diversity and different responses to the topic guide. In addition, there were significantly larger groups of patients with breast cancer (*n* = 9) and those from Syria (*n* = 11) interviewed than the other groups, which has limited the ability to explore and compare the perspectives from different groups.

### Implications

Universal Health Coverage states palliative care as an essential health service.^33^ In the WHO Global Action Plan for the Prevention and Control of Non-communicable Diseases 2013−2020,^34^ palliative care is explicitly recognised as part of the comprehensive services required for the non-communicable diseases. Palliative care and pain relief are among the most neglected dimensions of global health. Addressing the specific concerns of refugee populations with advanced cancer may achieve the goals of The Lancet Commission on Palliative Care and Pain Relief to alleviate the burden of pain, suffering and severe distress associated with life-threatening or life-limiting conditions.^35^

The capacity to meet the cancer and palliative care needs of refugees is limited. Capacity building and training would be the first step to improve cancer and palliative care for this vulnerable and neglected population in Jordan. Nurses from oncology departments in Jordan have described the working environment is highly stressful and demanding and they faced many challenges in their work, including the lack of authority to inform patients about their disease, nursing staff and supply shortages and a lack of orientation programmes.^36^ All these factors affected the psychological status of the nurses which would subsequently influence the quality of care they provided, therefore, should be taken into consideration by stakeholders, managers and organisational leaders.

## Conclusion

Referral networks and specialised training for humanitarian teams and health care providers in Jordan on palliative care such as implementing pain management, offering psychosocial support services and addressing emotional, spiritual and psychological conditions could ameliorate many of the problems faced by this vulnerable group. Community-based palliative care services should be enhanced in Jordan to meet the needs of this refugee population. Further research is required to explore the understanding of true experiences and needs of refugees with advanced cancer from healthcare providers’ perspectives and to compare the experiences of refugees with advanced cancer and their family caregivers from different social and cultural backgrounds.
